# Frequency and clinical impact of retained implantable cardioverter defibrillator lead materials in heart transplant recipients

**DOI:** 10.1371/journal.pone.0176925

**Published:** 2017-05-02

**Authors:** Jun Kim, Jongmin Hwang, Jin Hee Choi, Hyo-In Choi, Min-Seok Kim, Sung-Ho Jung, Gi-Byoung Nam, Kee-Joon Choi, Jae Won Lee, You-Ho Kim, Jae-Joong Kim

**Affiliations:** 1 Department of Cardiology, Asan Medical Center, University of Ulsan College of Medicine, Seoul, Korea; 2 Thoracic and Cardiovascular Surgery, Asan Medical Center, University of Ulsan College of Medicine, Seoul, Korea; Nagoya University, JAPAN

## Abstract

End-stage heart failure patients with implantable cardioverter-defibrillator (ICD) with/without cardiac resynchronization therapy (CRT-D) often require heart transplantation (HTPL) as a last-resort treatment. We aimed to assess the frequency and clinical impact of retained ICD lead materials in HTPL patients. In this retrospective single center study, we examined the clinical records and chest radiographs of patients with ICD and CRT-D who underwent HTPL between January 1992 and July 2014. Of 40 patients with ICD and CRT-D at HTPL, 19 (47.5%) patients had retained ICD lead materials within the central venous system. Retained ICD lead materials following HTPL were more frequently noted in patients with longer implantation durations until HTPL. None of the patients underwent extraction procedures after HTPL. All patients were asymptomatic and did not exhibit significant complications or death related to the retained ICD lead materials. Seven (7/40, 17.5%) patients without any retained ICD lead materials underwent magnetic resonance imaging (MRI) during the follow-up period (median, 29.5 months); none of the patients with retained lead materials were given MRI. Considering the common use of MRI in HTPL patients, further studies on the prophylactic extraction of retained ICD lead materials and safety of MRI in these patients are needed.

## Introduction

Implantable cardiac devices are used in advanced heart failure patients to improve the symptom, and prognosis. Implantable cardioverter-defibrillators (ICD) can prevent sudden cardiac death attributed to ventricular tachyarrhythmia, and cardiac resynchronization therapy (CRT), usually with defibrillation capability (CRT-D), can reduce symptoms and hospitalizations due to ventricular dyssynchrony in these patients [1–3]. Despite the beneficial effects of this therapy, end-stage heart failure patients often require heart transplantation (HTPL) because it is the regarded as the last-resort treatment option in these patients, and is associated with excellent long-term survival [4, 5]. The implanted ICD/CRT-D is usually removed at the time of HTPL operation. The generator is removed from the pocket and the portion of the leads dwelling within the atrium, ventricle, and lower part of the superior vena cava (SVC) are removed with the excision of the recipient’s heart. However, the leads within the central venous system can be difficult to remove without specialized extraction tools or techniques. This is particularly the case because ICD/CRT-D leads contain defibrillating coils. In this situation, tissue ingrowth and adhesion around coils act as binding points to the walls of large veins and hence complete endothelialization of lead materials into the vessel wall occurs more frequently [6]. As heart transplantation is never an elective procedure, it is not feasible to provide an additional team for lead removal, which might prolong the operation time. Evaluating whether lead materials have been completely removed requires fluoroscopic imaging of the procedural field, but surgical rooms are usually not equipped with an X-ray machine [7]. Therefore, complete removal of previously-implanted ICD/CRT-D during HTPL operation presents quite a clinical challenge, and, as a result, parts of the lead materials especially within the central venous system are often retained after the surgery [8].

Recently, indications for ICD/CRT-D have expanded, so the number of patients undergoing HTPL with implanted device is expected to increase. But the published literature regarding the frequency and clinical implications of retained ICD/CRT-D lead materials following HTPL is still lacking. The purpose of this study is to investigate the frequency of retained ICD/CRT-D lead materials in the central venous system after HTPL operation and to assess its clinical sequelae. In addition, we evaluated the proportion of patients who underwent magnetic resonance imaging (MRI) examination, which is one of the most important clinical issues in these patients due to the possible hazardous complications by retained lead materials [9].

## Methods

### Study patients

From November 1992 to December 2014, 474 consecutive patients underwent orthotopic HTPL at Asan Medical Center, Seoul, South Korea. Of these, 40 patients with ICD or CRT-D *in situ* at the time of HTPL were included in this study. Baseline demographic, historical, and procedural data were obtained from electronic medical records.

### HTPL operation, postoperative management, and follow-up

Prior to January 1999, standard anastomosis was performed. However, since then, all HTPL procedures have involved bicaval anastomosis. The details of the HTPL surgical procedures have been described previously [10]. At the time of HTPL operation, only manual traction from the implant site was made to remove the leads, with no further attempt to extract using extraction tools such as a locking stylet, mechanical sheaths, or snares. After the operation, all recipients were managed according to protocols that included immunosuppression, infection prophylaxis, endomyocardial biopsy, and coronary angiography; the details of these procedures have been described previously [5]. All HTPL patients received regular clinical follow-up at Asan Medical Center.

### Determination of retained lead materials

Chest radiographs were obtained in all patients after HTPL, and were reviewed independently by 2 cardiac electrophysiologists for the presence of retained ICD/CRT-D lead materials. Retained materials were classified based on the extent of remnants in the central venous system, and were classified into those localized to the SVC, those exist from SVC to left or right brachiocephalic vein, and those exist from SVC to left or right subclavian vein.

### Assessment of clinical implications

We retrospectively reviewed the data of study patients with focus on the following topics to assess the clinical implications: (1) any subsequent procedure performed to remove the retained lead not removed during HTPL, (2) any infection related to the retained lead, (3) evidence of embolization or erosion of the retained lead materials, and (4) mortality. In addition, the frequency and sites of the MRI examination after HTPL regardless of whether it was performed at our institution or not, were also investigated.

### Statistical analysis

SPSS version 18.0 (SPSS Inc., Chicago, IL) was used for all statistical analyses. Continuous variables are presented as means (±SD) or medians (quartiles, Q1–Q3) and categorical variables as numbers and percentages. Continuous variables were compared using the t-test or Mann–Whitney U test, and categorical variables were compared using χ2 statistics or Fisher's exact tests. A p value of <0.05 was considered statistically significant.

### Ethics statement

The study protocol was reviewed and approved by the institutional review board of Seoul Asan Medical center (IRB No. 2015–0068), which waived the requirement for informed consent based on the retrospective nature of the study. None of the transplant donors were from a vulnerable population and all donors or next of kin provided written informed consent that was freely given.

## Result

### Clinical characteristics of the study population

Of 474 patients who underwent heart transplantation (HTPL) at our institution, 40 patients had ICD or CRT-D *in situ* at the time of HTPL. The mean age was 50±9.63 years, and 30 patients (75%) were male. The underlying cardiac diseases that led to the HTPL included dilated cardiomyopathy in 27 patients (67.5%), ischemic cardiomyopathy in 7 patients (17.5%), and other causes in 6 patients (15%). Among 40 patients, 35 patients (87.5%) had an ICD, whereas 5 patients (12.5%) had a CRT-D. The median duration between ICD/CRT-D implantation and HTPL was 30 months (17–62 months), and the median number of leads was 2 (1–4).

Nineteen out of 40 patients (47.5%) had retained ICD/CRT-D lead materials within the central venous system after HTPL, as confirmed by postoperative chest radiography. The clinical characteristics of those with and without retained lead materials are shown in Table 1 (Individual-level data are presented in the S1 Table). Patients with retained lead materials had a longer interval from device implantation to HTPL than those without (p = 0.01).

**Table 1:** Baseline characteristics of patients at the time of heart transplantation.

	Overall (n = 40)	With retained ICD/CRT-D lead materials (n = 19)	Without retained ICD/CRT-D lead materials (n = 21)	p value
Age at heart transplantation, years	51.6±9.6	52.3±9.5	50.9±9.9	0.650
Male, n (%)	30 (75%)	15 (78.9%)	15 (71.4%)	0.583
BMI	22.8±4.2	22.6±3.7	22.9±4.7	0.830
Etiology, n (%)				
Dilated CMP	27 (67.5%)	11 (57.9%)	16 (76.2%)	0.314
Ischemic CMP	7 (21.1%)	4 (21.1%)	3 (14.3%)	0.574
Hypertrophic CMP	3 (7.5%)	3 (15.8%)	0 (0%)	0.058
Others	3 (7.5%)	1 (5.3%)	2 (9.6%)	0.335
History of AF, n (%)	14 (35.0%)	9 (47.4%)	5 (23.8%)	0.186
History of ventricular arrhythmia, n (%)	31 (77.5%)	17 (89.5%)	14 (66.7%)	0.085
Type of device, n (%)				
ICD	35 (87.5%)	18 (94.7%)	17 (81.0%)	0.188
CRT-D	5 (12.5%)	1 (5.3%)	4 (19.0%)	0.188
Type of defibrillator lead, n (%)				0.335
Single coil	1 (2.5%)	0 (0%)	1 (4.8%)	
Dual coil	39 (97.5%)	19 (100%)	20 (95.2%)	
Number of leads at the time of heart transplantation, median (range)	2 (1–4)	2 (1–3)	2 (1–3)	0.415
Duration from device implant to heart transplantation, median (range), months	26 (1–154)	31 (18–154)	18 (1–146)	0.010

Data are presented as mean ± SD, median (interquartile range), or number (%). Abbreviations: BMI, body mass index; CMP, cardiomyopathy; AF, atrial fibrillation; ICD, implantable cardioverter defibrillator; CRT-D, cardiac resynchronization therapy (CRT) with defibrillation capability.

### Retained ICD/CRT-D lead materials

Table 2 describes the nature of the retained ICD/CRT-D lead materials and their extent (Individual-level data are presented in the S1 Table). All of the lead materials retained in the central venous system involved defibrillator coil, with or without lead. The lead materials were retained in SVC only in 7 patients (7/19, 36.8%), retained from SVC to left or right brachiocephalic vein in another 7 patients (7/19, 36.8%), and retained from SVC to left or right subclavian vein in 5 patients (5/19, 26.3%).

**Table 2:** Characteristics of the retained ICD/CRT-D lead materials following heart transplantation.

Components of retained lead materials, n (%)	
Coil	14 (73.7%)
Lead	0 (0%)
Coil and lead	5 (26.3%)
Extent of the retained lead materials, n (%),	
SVC	7 (36.8%)
From SVC to justify or Right brachiocephalic vein	7 (36.8%)
From SVC to justify or Right subclavian vein	5 (26.3%)

Data are presented as number (%). Abbreviations: ICD, implantable cardioverter defibrillator; CRT-D, cardiac resynchronization therapy (CRT) with defibrillation capability; SVC, superior vena cava.

### Clinical sequelae of retained ICD/CRT-D lead materials

During the follow up period, no subsequent procedure was performed to remove the retained lead materials not removed during HTPL. Infection related to the retained lead and embolization or erosion of the retained lead materials did not occur. The overall mortality rate was 7.5% (3/40) during the study period. In one patient with retained lead materials, the cause of death was pneumonia-related sepsis. In two patients without retained lead materials, lung cancer and sepsis due to opportunistic infection were the cause of death, respectively.

### MRI examination after HTPL

Of 21 patients without retained ICD/CRT-D lead materials, 7 patients were examined by MRI. The sites of the MRI scan were as follows: brain in 12, lumbar spine in 1, and liver in 1 patient. None of the patients with retained ICD/CRT-D lead materials underwent MRI during the follow-up period. Of 474 patients who underwent HTPL at our institution from January 1992 to July 2014, 168 underwent 308 MRI procedures during the follow-up period. The sites of the examinations are described in Table 3 (Individual-level data are presented in the S2 Table).

**Table 3:** MRI examination in HTPL patients.

	Patients without ICD/CRT-D at HTPL (n = 434)	Patients without remnant ICD/CRT-D lead materials after HTPL (n = 21)	Patients with remnant ICD/CRT-D lead materials after HTPL (n = 19)
Number of patients who underwent MRI	168 (38.7%)	7 (17.5%)	0
Total number of MRI procedures	294	14	0
Site of MRI			0
Brain	211	12	0
Lumbar spine	25	1	0
Lower extremities	17	0	0
Hip	15	0	0
Abdomen	12	1	0
Cervical spine	9	0	0
Upper extremities	4	0	0
Heart	1	0	0

Data are presented as number or number (%). Abbreviations: MRI, magnetic resonance imaging examination; HTPL, heart transplantation; ICD, implantable cardioverter defibrillator; CRT-D, cardiac resynchronization therapy (CRT) with defibrillation capability.

## Discussion

In the present study, we observed that 47.5% (19/40) of patients who had ICD/CRT-D at the time of HTPL had retained ICD/CRT-D lead materials within their central venous system. No subsequent procedures were undertaken to remove retained lead materials, and there were no retained lead materials related complications such as lead fragment embolization, migration, erosion, and infection. All patients who had retained lead materials were asymptomatic and did not display adverse clinical sequelae. None of these patients were permitted to undergo MRI.

Despite advances in techniques and tools for transvenous lead extraction, the extraction of defibrillator leads including coils remains challenging, and is associated with minimal, but significant, mortality [11, 12]. The coils of ICD leads induce extensive growth of scar tissue, which surrounds and entraps the lead; hence, complex extraction procedures are required [13, 14]. A previous study showed that areas of adherence of the ICD lead were detected in the subclavian vein (78%), brachiocephalic vein (65%), SVC (66%), and heart (73%) [8]. Dwell time, passive fixation, and dual-coil lead design were independently associated with adherence [8]. Dual coil ICD is a risk factor for extraction difficulty, and is associated with SVC rupture [15]. In the present study, patients with retained ICD/CRT-D lead materials showed longer interval from device implantation to HTPL than those without. This finding is consistent with that in previous studies, wherein long dwell time was found to be associated with the need for advanced tools during ICD extraction [16–18] and lead extraction failure during HTPL [6].

Non-functioning leads themselves may be associated with a small risk of complications such as infection, venous occlusion, lead migration, and skin erosion [19–22]. In the present study, there were no long-term sequelae related to retained ICD/CRT-D lead materials, such as lead-related infection, thrombosis, vascular obstruction, and embolization. The absence of vascular obstruction can be attributed to the small number of ICD/CRT-D lead(s) (1 to 2) in each patient, because the risk of vascular obstruction is positively related to the number of retained leads. Moreover, the absence of ICD/CRT-D lead material embolization can be attributed to severe fibrosis around the SVC coil. These data are consistent with the results of previous studies on patients with and without HTPL [6, 23]. Considering that the presence of abandoned ICD/CRT-D leads is not associated with risks to patients, abandoning the retained lead materials may represent a reasonable strategy, particularly in institutions where laser sheaths are not available [23, 24]. However, recent data have suggested the feasibility and safety of lead extraction after HTPL using laser sheath and snares [7].

HTPL has evolved into a treatment modality for end-stage heart failure. Although this method is associated with excellent long-term survival, graft vasculopathy, infection, and malignancy are the leading causes of death [4]. Moreover, MRI is an indispensable clinical diagnostic tool for the diagnosis of cardiac and non-cardiac diseases due to the excellent spatial resolution and multiplanar 3-dimensional analysis, as well as the absence of risk of exposure to ionizing radiation and potentially nephrotoxic iodinated contrast agents [25–28]. In our heart transplant cohort, 35.4% (168/474) of patients required MRI for various reasons. The most common examination sites included the brain (72.4%), spine (11.3%), and extremities (6.8%). During the follow-up duration of 30 months, there were no difference in clinical outcome between patients with and without retained ICD/CRT-D lead materials; however, it should be noted that a greater need for MRI in heart transplant recipients increases management issues related to these retained lead materials. A remnant nonfunctional lead is a contraindication for MRI due to the potential hazard for patients [29, 30]; this supports practicing routine extraction of retained lead materials in HTPL recipients. Although previous case series have suggested the safety of MRI in patients with retained leads [6, 9], experimental data have indicated the potential harm of MRI in patients with abandoned pacemaker leads [31, 32]. Nevertheless, there is a paucity of data on the hazards of MRI, particularly in cases with retained ICD/CRT-D lead materials.

### Limitations

Our current study is partially and inherently limited in that it is a retrospective investigation. However, to our knowledge, this is the largest study on heart transplant recipients with retained ICD/CRT-D lead materials in Asia; for this reason, we believe that our study holds considerable value for clinicians. Second, the use of advanced extraction tools might continue to diminish this clinical dilemma of retained lead. However, considering the advanced disease stage of the recipients, the emergency nature of HTPL, and the potential risk in lead extraction procedure, it is possible that lead extraction with advanced extraction tools should not be performed simultaneously or after HTPL. Third, the overutilization of MRI in these cases has been suggested. Our current study reflects the real-world practice of a dedicated heart transplant care team. Considering the various post-HTPL complications, MRI examination is essential in caretaking of HTPL patients, especially to exclude central nervous system infection and to evaluate spine or joints disorders. However, due to the retrospective nature of our analysis, we could not assess the appropriateness of the MRI examination in all the patients.

### Conclusion

Advanced heart failure patients require implantable cardiac devices such as ICD/CRT-D to improve their disease status and prognosis. Some of those patients, however, eventually require HTPL at the end stage of heart failure as a last-resort treatment. These implanted devices are usually removed at the time of HTPL operation. However, the defibrillating lead coils within the central venous system are difficult to be completely removed due to adhesion to host tissue. Therefore, ICD/CRT-D lead materials are frequently retained in the central venous system of HTPL patients. Although there were no clinical sequelae related to retained ICD/CRT-D lead materials in our study patients, the greater need for MRI in HTPL patients warrant further studies geared toward recommending routine extraction of retained lead materials for the safety of MRI procedure in this group of patients.

## Supporting information

S1 Table Individual-level data underlying results presented in the Tables 1 and 2. (XLSX)

S2 Table Individual-level data underlying results presented in the Table 3. (XLSX)
